# Fast Parabolic Fitting: An R-Peak Detection Algorithm for Wearable ECG Devices

**DOI:** 10.3390/s23218796

**Published:** 2023-10-28

**Authors:** Ramón A. Félix, Alberto Ochoa-Brust, Walter Mata-López, Rafael Martínez-Peláez, Luis J. Mena, Laura L. Valdez-Velázquez

**Affiliations:** 1 Facultad de Ingeniería Mecánica y Eléctrica, Universidad de Colima, Colima 28400, Mexico; rfelix@ucol.mx; 2 Departamento de Ingeniería de Sistemas y Computación, Universidad Católica del Norte, Antofagasta 1249004, Chile; rafael.martinez@ucn.cl; 3 Unidad Académica de Computación, Universidad Politécnica de Sinaloa, Mazatlán 82199, Mexico; lmena@upsin.edu.mx; 4 Facultad de Ciencias Químicas, Universidad de Colima, Colima 28400, Mexico; lauravaldez@ucol.mx

**Keywords:** R-peak detection, fast parabolic fitting, wearable ECG devices

## Abstract

Heart diseases rank among the most fatal health concerns globally, with the majority being preventable through early diagnosis and effective treatment. Electrocardiogram (ECG) analysis is critical in detecting heart diseases, as it captures the heart’s electrical activities. For continuous monitoring, wearable electrocardiographic devices must ensure user comfort over extended periods, typically 24 to 48 h. These devices demand specialized algorithms with low computational complexity to accommodate memory and power consumption constraints. One of the most crucial aspects of ECG signals is accurately detecting heartbeat intervals, specifically the R peaks. In this study, we introduce a novel algorithm designed for wearable devices, offering two primary attributes: robustness against noise and low computational complexity. Our algorithm entails fitting a least-squares parabola to the ECG signal and adaptively shaping it as it sweeps through the signal. Notably, our proposed algorithm eliminates the need for band-pass filters, which can inadvertently smooth the R peaks, making them more challenging to identify. We compared the algorithm’s performance using two extensive databases: the meta-database QT database and the BIH-MIT database. Importantly, our method does not necessitate the precise localization of the ECG signal’s isoelectric line, contributing to its low computational complexity. In the analysis of the QT database, our algorithm demonstrated a substantial advantage over the classical Pan-Tompkins algorithm and maintained competitiveness with state-of-the-art approaches. In the case of the BIH-MIT database, the performance results were more conservative; they continued to underscore the real-world utility of our algorithm in clinical contexts.

## 1. Introduction

The electrocardiogram (ECG) allows for the convenient measurement of cardiac electrical activity. Successive waveforms, known as the P wave, QRS complex, and T wave, characterize each cardiac cycle on the ECG. These waveforms represent the depolarization and repolarization activities in the cells of the atrium and ventricle. Automatic detection of ECG waveforms provides essential information for diagnosing cardiac disease. This broad field continues to be an active research topic, as evidenced by recent publications [1,2]. Due to the significant morphological variation of ECG signals, it is not easy to design automatic and widely applicable algorithms. This difficulty partly explains the continuous effort made by researchers in ECG signal processing. ECG detection algorithms aim to improve classification accuracy and are becoming as reliable and successful as expert cardiologists.

Researchers have extensively investigated automatic R-peak detection as a classic ECG signal processing problem. The well-known real-time R-peak detection algorithm was proposed by Pan-Tompkins [3]. This classical algorithm can process and display the detection result for each sample after a learning period. However, the complexity of the Pan-Tompkins algorithm is high, and the detection accuracy is moderate compared with recently developed algorithms [1,4,5].

Several notable studies have recently been conducted [4,6,7]. However, a problem with most R-peak detection algorithms is that the computational complexity could be higher, and they require impractical assumptions, e.g., they usually need a global statistical knowledge of the entire input signal [3,8,9,10,11].

On the other hand, researchers have focused on developing efficient sensing algorithms suitable for wearable ECG devices [5,12,13,14,15]. The authors proposed a simple real-time R-peak detector with low computational cost [16]. As in many previous works, the authors of [15] customized the input parameters of the algorithm for optimal performance on the MIT-BIH arrhythmia database, which may not be suitable for other databases. Moreover, it is not robust to noise. According to the comprehensive review by the authors, several studies are mentioned in Chapter 4 that achieve a transcendental level of success in determining the R peak. Nevertheless, the algorithm’s performance is susceptible to heart rate variations.

Researchers have successfully used two-parameter parabolic fitting to detect R points of ECG signals [1,7]. However, these methods are not suitable for wearable devices since they use computationally complex processes such as high-order band-pass filters, isoelectric line localization, and wavelet filtering. Additionally, one needs a comprehensive understanding of the complete ECG signal.

The proposed method utilizes single-parameter parabolic fitting to extract a feature known as parabolic height. This feature detects partial P peaks, or candidates, by comparing them with a parabolic height threshold. Then, the best candidates are designated as R points of the ECG signal. Afterwards, this threshold is adapted to different signal amplitudes. The proposed algorithm does not require calculating and estimating the isoelectric line of the ECG signal, nor does it require any prior filtering. The authors tested the algorithm on two databases, QT and BIH-MIT. The results show that the proposed algorithm performs very well compared to previous work in terms of robustness to noise and reduced computational resources. It is essential to highlight that the algorithm presented in this document improves the performance of the previous algorithm, called LDR, by reducing the number of operations and the performance of the detection of maximum and minimum points.

## 2. Materials and Methods

This paper proposes a novel algorithm for R-peak detection, considering the limitations of computational resources commonly found in wearable ECG devices. We tested the algorithm for detecting R peaks in ECG signals from two widely used research databases: the QT database and BIH-MIT database. These ECG signals were obtained following ethical guidelines, as reported in [17,18,19,20]. We conducted all programming for this work on the MATLAB platform.

The QT database contains a total of 105 15 min ECGs. We chose the ECGs in this database to represent various QRS and ST-T morphologies with real-world variability, aiming to challenge detection algorithms [18]. The sampling rate in this database is 360 Hz, allowing for a total of 86,995 beats from 82 recordings to be stored, and the remaining 23, sel30-sel52, were not considered due to the absence of QRS annotations. This number is consistent with the results of other studies [5].

The BIH-MIT database contains 48 ECG records, each containing a 30 min ECG signal [19]. They used the entire dataset, totaling 109,490 beats. Twenty-five recordings contain fewer common arrhythmias. The recordings have a sampling rate of 360 Hz with a resolution of 11 bits at a 10 mV interval. The ECG recordings have acceptable quality, high and sharp P and T waves, negative R waves, small R peak amplitudes, wider R waves, muscle noise, deviation from baseline, sudden changes in beat morphology, multiform PVCs, long pauses, and irregular heart rhythms.

This work ignores all non-beat annotations defined by Physionet [19]. Note that both databases offer two channels of ECG signals. This study used only the first channel to develop and test the algorithm.

To be consistent with most of the R-peak detection studies [3,5,8,9,10,11,12,14,15], we used quantitative comparisons in terms of sensitivity (S), positive predictability (P), and detection error (DER), as defined in Equations (1)–(3):

(1) $$S\left(\%\right)=\frac{TP}{TP+FN}$$

(2) $$P\left(\%\right)=\frac{TP}{TP+FP}$$

(3) $$\mathrm{DER}\left(\%\right)=\frac{FN+FP}{TP+FP}$$

The true positive (TP) is defined as the number of R peaks detected, false negative (FN) is the number of true R peaks that was not detected, and false positive (FP) is the number of detected R peaks that is not detected. Sensitivity represents the percentage of true beats correctly detected, whereas positive predictability presents the percentage of true beats detected.

A problem with the BIH-MIT and QT databases is that the beat annotations are often not located at peak R. This poses a severe problem when evaluating the detectors, as it introduces a temporal fluctuation in the time stamps of peak R. For this reason, we made a readjustment to the original annotations. After applying the algorithm to each recording, they automatically calculated the TP, FN, and FP indices using the new beat annotations, with a tolerance of 40 ms [20]. This tolerance is consistent with similar work, although sometimes implicit.

## 3. Fast Parabolic Fitting for ECG Signals

Parabolic fitting aims to extract from a neighborhood of a point in the ECG signal a feature that indicates that the point is the R point and that the neighborhood is part of the R peak. Parabolic fitting has already been successfully applied in the analysis of ECG signals [1,17]. One of the significant advantages of parabolic fitting is that it is not necessary to calculate the isoelectric line of the ECG signal. However, in such schemes, a parabola fitting uses two parameters: window width and parabola height, increasing the computational cost and affecting the performance of wearable devices. The computational cost increases by the number of additional operations required, such as 6L+2 additions, 2L+4 multiplication, and 2 divisions.

**Figure 1:**
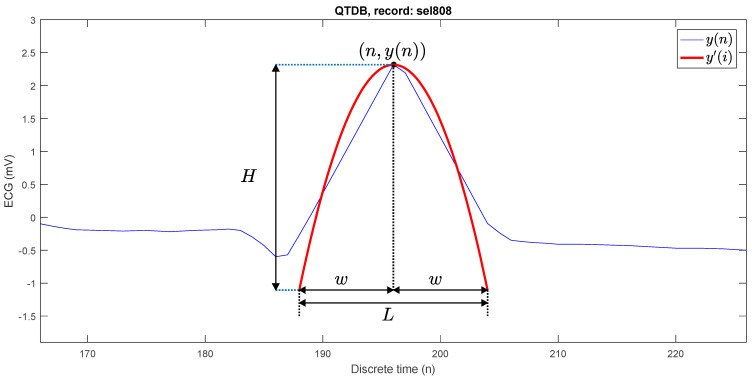
Parabola fitting at a P peak.

In response to the prior inconveniences, we have introduced a novel approach to parabolic fitting, employing only one parameter. This modification substantially reduces the number of addition and multiplication operations, effectively improving the computational cost, a critical factor in enhancing the performance of wearable devices. Furthermore, our approach eliminates division operations, resulting in a more efficient and streamlined process. Figure 1 shows the parabolic approximation at an R peak.

For each point (n,y(n)) of the ECG signal, the best fitting parabola has the following form:

(4) $$y^{\prime }\left(i\right)=a{\left(n-i\right)}^{2}+y\left(n\right).$$

where i∈{n−w,…,n−1,n,n+1,…,n+w}, *y*(*n*) is the original ECG signal, *a*(*n* − *i*)^2^ is the parabola, and *y′*(*n*) is the adjustment of the signal ECG with the fitting parabola.

This adjustment in the equation allows us to extract from a neighborhood of values the representative values that can be adjusted to a maximum or minimum value in the analysis window, allowing us to improve detection and ensure that detection is successful. The length of a window is

(5) L=2w+1.

The polynomial coefficient a is calculated by minimizing the following quadratic error criterion:

(6) $$V(a)=\frac{1}{2}\overset{n+w}{\underset{i=n-w}{\sum }}{e\left(i\right)}^{2}$$

The first derivative of V(a) is computed as

(7) $$\frac{\partial V\left(a\right)}{\partial a}=\overset{n+w}{\underset{i=n-w}{\sum }}e\left(i\right)\frac{\partial e\left(i\right)}{\partial a}=\overset{n+w}{\underset{i=n-w}{\sum }}\left(y\prime \left(i\right)-y\left(i\right)\right)\frac{\partial e\left(i\right)}{\partial a}.$$

Since

(8) $$\frac{\partial e\left(i\right)}{\partial a}=\frac{\partial y\prime \left(i\right)}{\partial a}={\left(n-i\right)}^{2},$$

The error (i)= y′(i)−y(i), therefore

(9) $$\frac{\partial V\left(a\right)}{\partial a}=\overset{n+w}{\underset{i=n-w}{\sum }}\left(a{\left(n-i\right)}^{2}+y\left(n\right)-y\left(i\right)\right){\left(n-i\right)}^{2}.$$

To minimize the criterion of Equation (4), the following equation must be solved:

(10) $$0=\overset{n+w}{\underset{i=n-w}{\sum }}\left(a{\left(n-i\right)}^{2}+y\left(n\right)-y\left(i\right)\right){\left(n-i\right)}^{2}.$$

Solving (10) for a, it yields

(11) $$0=\overset{n+w}{\underset{i=n-w}{\sum }}a{\left(n-i\right)}^{2}+y\left(n\right)-y\left(i\right)$$

(12) $$\overset{n+w}{\underset{i=n-w}{\sum }}y\left(i\right)-y\left(n\right)=a\overset{n+w}{\underset{i=n-w}{\sum }}{\left(n-i\right)}^{2}$$

(13) $$\frac{\sum_{i=n-w}^{n+w}y\left(i\right)-y\left(n\right)}{\sum_{i=n-w}^{n+w}{\left(n-i\right)}^{2}}=a.$$

Given that $\sum_{i=n-w}^{n+w}{\left(n-i\right)}^{2}$ is constant for any y(n)

(14) $$C=\frac{1}{\sum_{i=n-w}^{n+w}{\left(n-i\right)}^{2}}$$

and

(15) $$\overset{n+w}{\underset{i=n-w}{\sum }}y\left(i\right)-y\left(n\right)=-Ly\left(n\right)+\overset{n+w}{\underset{i=n-w}{\sum }}y\left(i\right).$$

Equation (13) is simplified

(16) $$a=C\left(-Ly\left(n\right)+\overset{n+w}{\underset{i=n-w}{\sum }}y\left(i\right)\right).$$

Calculation for the coefficient a at each sample of the ECG signal requires L+1 additions and 2 multiplications, which makes this algorithm more suitable for implementation on low-cost microprocessors. On the other hand, if a is negative, it indicates that the peak is upward, and if positive, the peak is downward. The parabola defined with the optimal parameter of Equation (16) is called the best-fit parabola.

The parabolic height (H) is defined as the absolute of the difference between the highest point and the lowest point of the best-fit parabola, which is computed as shown in (17) and this value *H* is used to detect the R peaks:

(17) $$H=\left|a\right|w^{2}.$$

**Figure 2:**
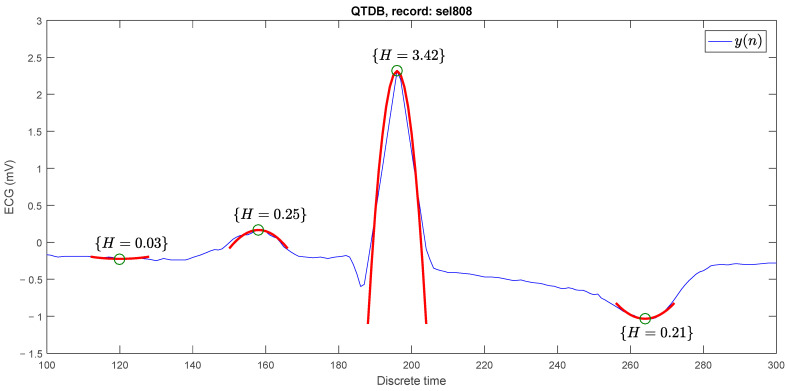
Parabolic approximations (red parabolas) in different points (green circles) of the ECG signal with different parabolic heights.

Figure 2 shows four parabolic approximations at different discrete time instants: n=158, n=186, n=196, and n=265. In the first case H=0.03, it is easy to see that there is no peak. In the second case, the P wave is present with H=0.25. In the third case, it does correspond to an R peak with H=3.42. In the fourth case it is H=0.25, even though the peak is downward. The natural strategy is to propose a threshold $H_{th}$, such that if $H>H_{th}$, then at time n there is an R peak. In each of the analysis windows, a value of *H* was obtained, where this coincides with the maximum value in height, and in the last point, it was established as a minimum value.

**Figure 3:**
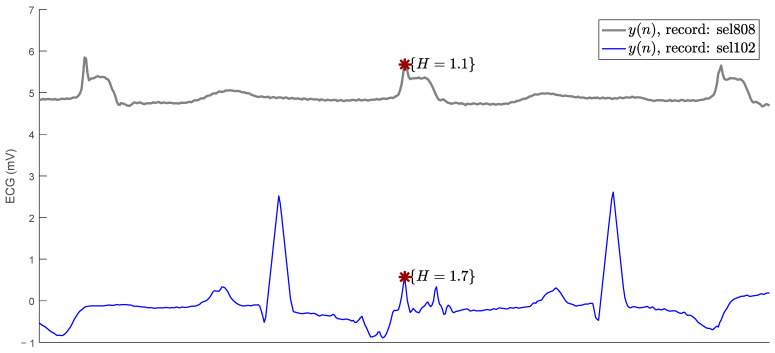
A true R peak (above red asterisk) and a false positive (below red asterisk) with higher parabolic height.

Figure 3 shows one of the biggest problems faced by R-peak detection algorithms in ECG signals. The figure shows an R peak in the sel808 recording and a noise peak in the sel102 recording. Both signals, from the QT database, have different isoelectric lines. The R peak has a parabolic height of H=1.1 and the false peak is higher with H=1.6. Under the threshold strategy of $H_{th}=1.5$, the false peak would result in a false positive and the R peak would result in a false negative. The need arises for $H_{th}$ to be variable, depending on the typical amplitude of the R peaks in the signal.

## 4. Fast Parabolic Fitting Algorithm R Peak for Detection

**Figure 4:**
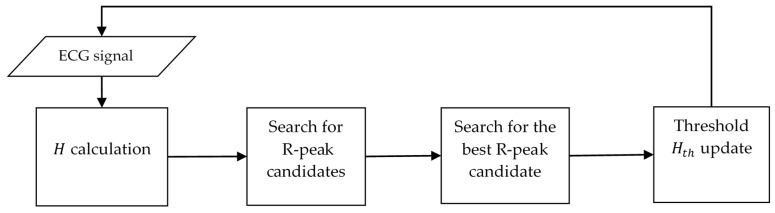
The proposed algorithm structure.

In general, the algorithm consists of four processes: H calculation for each sample, searching for R peak candidates, searching for the best candidate for R peak, and adaptation of the parabolic height threshold ($H_{th}$), see Figure 4.

Each ECG signal sample has its parabolic height (H) calculated, and if $H>H_{th}$, then that time is defined as an R-peak candidate. If a candidate has been the best candidate for a certain time, it is recorded as an R peak, then the $H_{th}$ threshold is updated.

The search for the best candidate starts when the first candidate is found and ends when a discrete time $n_{cand}$ elapses without the best candidate being replaced. For an ECG signal point to be detected as an R peak, it must first be detected to pass as a candidate, then as a best candidate, and remain as such for $n_{cand}$ samples. If a signal R peak does not pass any of these three stages, it will be discarded, which will cause the number of false positives to increase.

**Figure 5:**
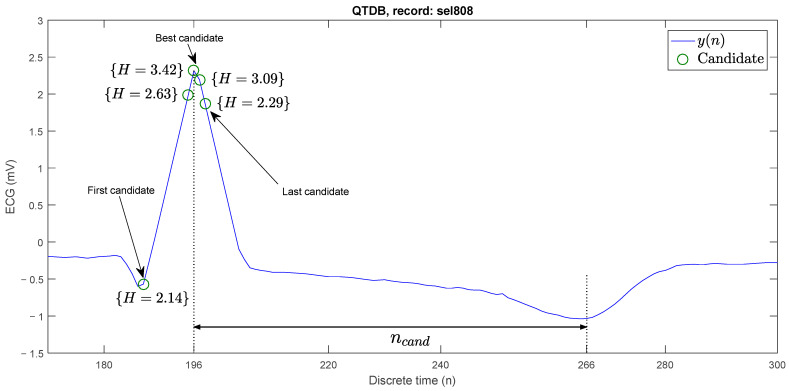
Search for the best R-peak candidate on an ECG signal.

For instance, with a threshold of $H_{th}=2.1$, Figure 5 shows five candidates at times: n=187, n=195, n=196, n=197, n=198. At time $n=196+n_{cand}$, the search for the best candidate ends, and it is designated that at n=196, there is an R peak.

In ECG signals, there is a large variability in the amplitude of R peaks; therefore, it is not possible to select a fixed value for $H_{th}$. If the $H_{th}$ threshold is quite large, more points with low parabolic height will qualify as candidates and it becomes more likely that the number of false positives will increase. Conversely, if the $H_{th}$ threshold is quite small, a true R peak with parabolic height less than the threshold will not qualify as a candidate, thus increasing the number of false negatives. Therefore, the way the threshold is adapted is crucial for the performance of the algorithm.

The threshold $H_{th}$ is adapted using a weighted arithmetic mean of the parabolic heights of the last M detected R peaks.

### 4.1. Pseudo Codes for the Proposed R-Peak Detection Algorithm

The proposed algorithm is described, in detail, by means of pseudo code in Algorithm 1. A discrete time window of width L=2w+1 moves along the ECG signal. At each time n, the parabolic height H of the signal is calculated. When the height H is greater than a threshold $H_{th}$, at time n there is an R-peak candidate. Once the first candidate is detected, the algorithm searches for the best candidate in a variable interval, called the candidate interval. The candidate search ends after a time $n_{cand}$, when no other best candidate has been found. Similarly, the threshold value $H_{th}$ is updated, by means of an average of the heights of the last M R peaks that have been detected. With this strategy, the threshold is adapted to the parabolic heights of the R peaks of the various ECG signals. This average is weighted by the constant parameter $\alpha_{H}$. If a time $n_{th}$ elapses without finding a new candidate after the last R peak was found, then the value of $H_{th}$ is reduced by adding the minimum value $H_{min}$, as if an R peak with parabolic height $H=H_{min}$ had been found and then averaged. This process is repeated every $n_{th}$ that no new R peak is found. $n_{s}$ is the last discrete time that an R peak should be detected, but it was not. The last part of the algorithm comprises the guarantee that $H_{min}\leq H_{th}\leq H_{max}$.
**Algorithm 1:** Pseudo code for the R-peak detection proposed methodA window is moved along the signal**for** n=w  to n=N−wa is calculated at n
 $a\leftarrow C\left(-Ly\left(n\right)+\overset{n+w}{\underset{i=n-w}{\sum }}y\left(i\right)\right)$
*H* is calculated at n
 $H\leftarrow \left|a\right|w^{2}$
Check for a new candidate at n
 **if** (*H*$\;>\;H_{th}$)Flag for a found candidate  candidateFound ←
**true**
Check if the new candidate is the best one  **if** (*H*$\;>\;H_{best}$)Update the new best candidate location   $n_{best}\leftarrow n$
Update the new best candidate height   $H_{best}\leftarrow \;H$

  **end**

 **end**
Check for ending the peak search **if** $\;(n-n_{best}>n_{cand}$
**and** candidateFound==**true**)For restarting a new peak search  candidateFound ←
**false**
A new R peak is registered  R_Peaks ≪n
$H_{th}$ update for new R peak  $H_{th}\;\leftarrow \;\alpha_{H}$**M_mean**$(H_{best}$)For checking the absence of new R peaks   $n_{s}\;\leftarrow \;n_{best}$
 **end**
Check for absence of new R peaks **if** ($n-n_{s}>n_{th}$)$H_{th}$ decreasing for finding new R peaks  $H_{th}=\alpha_{H}$**M_mean**$(H_{min}$)For checking the absence of new R peaks   $n_{s}\leftarrow n$

 **end**
$\mathrm{Assuring}\;H_{min}\leq H_{th}\leq H_{max}$ $H_{th}\leftarrow \;$**saturation**$(H_{th}$)

Algorithm 2 describes the pseudo code to calculate the average of the last M P peaks detected by the algorithm. This function is also used to decrement the $H_{th}$ threshold when there are no new R peaks.
**Algorithm 2:** Pseudo code for the M_mean function
**M_mean**(*H*)Shifting the last *M* R-peak heights $h_{M}\leftarrow h_{M-1}$

 $h_{M-1}\leftarrow h_{M-2}$

 ⋮

 $h_{1}\leftarrow H$
Mean of R-peak heights $h\leftarrow \sum_{i=1}^{M}h_{i}/M$
**return** h

Algorithm 3 shows the pseudo code for limiting the $H_{th}$ threshold value.
**Algorithm 3:** Pseudo code for the saturation function
**saturation**(h)$\mathrm{Assuring}\;H_{min}\leq H_{th}\leq H_{max}$ **if** $(h<H_{min}$)
  $h\leftarrow H_{min}$

 **else if** $(h>H_{max}$)
  $h\leftarrow H_{max}$

 **else**

**return** *h*

The proposed algorithm has only eight numerical parameters: $H_{min}$, $H_{max}$, $\alpha_{H}$, $n_{cand}$, $n_{th}$, $n_{s}$, L, and M, that must be updated according to the different databases where it is tested.

### 4.2. Analysis of Computational Complexity

The proposed algorithm was implemented on a desktop computer in MATLAB language. However, for the implementation of the algorithm in wearable devices, it is necessary to program low-power microcontrollers or programmable logic devices such as FPGA. One way to compare the computational complexity of algorithms is very common to use the number of registers and operations of addition, multiplication, and comparison. Compared to some of the more advanced portable implementations (see Table 1), the proposed algorithm is competitive. The number of resources is similar or lower than the alternative proposals.

## 5. Results

### 5.1. Detection Results for the QT Database

The proposed algorithm was implemented in MATLAB. The numerical values of the parameters were defined by performing an exhaustive search. Table 2 shows the values that gave the best performance of the algorithm for the QT database.

Table 3 presents the performance of the proposed algorithm for the QT database. The detection error (DER) is 0.17%, the sensitivity (S) is 99.9%, and the positive predictability (P) is 99.88%. It is noteworthy that a total of 47 (57.3%) records had a DER of 0%, i.e., without any false negative or false positive. The values shown in Table 3 were calculated considering the entire dataset and no result was minimized.

**Figure 6:**
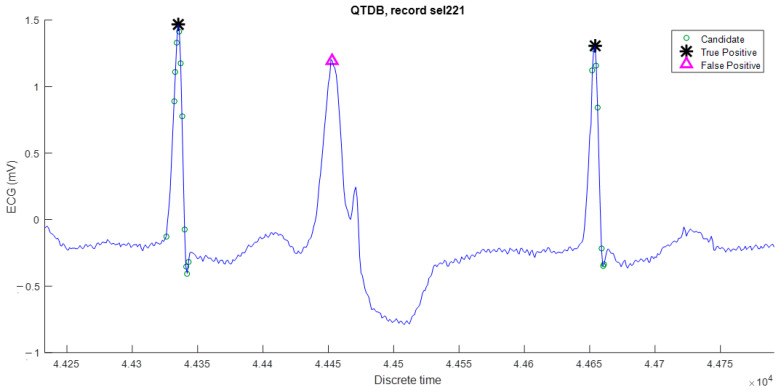
False negative is due to its reduced parabolic height.

The algorithm performs well on most records and its maximum DER is less than 1%, except for four records: sel308, sel847, sel15814, and sele0129. The false negative type is mostly due to the R peak being wider and lower than most of the R peaks, as is the case for the false positive present in Figure 6.

**Figure 7:**
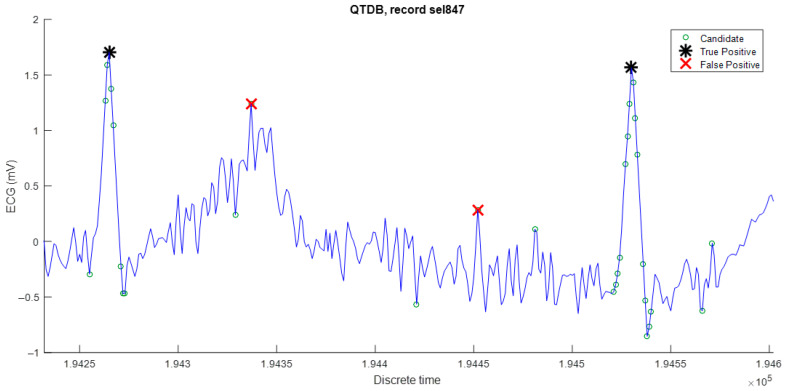
False positive R peaks provoked by high-frequency noise.

On the other hand, false positives are mainly due to high-frequency noise, as shown in Figure 7. This phenomenon could be reduced by using band-pass filtering, but it would increase the computational complexity of the algorithm. Despite this, the results are still very satisfactory for the QT database with respect to other algorithms published in the literature.

A comparison of the performance of the proposed algorithm with other algorithms is shown in Table 4. The proposed algorithm is superior to the others, except in positive predictability (P) by the algorithm of Pandit et al. [12].

### 5.2. Detection Results for the BIH-MIT Database

The sampling frequency in the BIH-MIT database is 360 Hz, and in the QT database, it is 250 Hz. This difference necessitates a readjustment of the algorithm parameters. Unfortunately, at this stage of the research, it was not possible to develop a methodology to readjust the parameters considering only the change in the sampling period; therefore, it was necessary to conduct the readjustment using brute force. This is because the BIH-MIT database has some records with higher levels of low frequency. In such records, false peaks are very similar to real ones, but closer to each other, which demanded a higher value for the parameter $\alpha_{H}$, and consequently, other parameters are needed to be readjusted. Table 5 shows the parameters of the proposed algorithm rescaled for the BIH-MIT database.

Table 6 presents the performance of the proposed algorithm against all records. The mean value of sensitivity (S) is 99.65%, the mean value of positive predictability (P) is 99.63%, and the mean value of error deviation (DER) is 0.67%. The algorithm works with acceptable efficiency, except for the following records: 108, 201, 203, 207, and 210, where it yields some false positives and negatives due to the high level of noise contained in these records.

The proposed algorithm is comparable with some outstanding ones in the literature for R-peak detection, as shown in Table 7, where the comparison of the proposed algorithm is shown.

### 5.3. Evaluating the Robustness to Noise of R-Peak Detection Algorithm

In the MIT-BIH arrhythmia database, there are many records, such as 121, 202, 200, 200, 217, 105, and 108, which are greatly affected by noise, including baseline drift and muscle noise. These records were used to evaluate noise robustness in some previous work [5,24]. Table 8 shows comparisons of the DER values of the proposed method with the other ten studies. The DER value of the proposed algorithm is comparable to that of previous works on the same records, which are heavily contaminated by noise.

In Bae [10], R-peak detection is tested in the presence of white noise. To records 102 and 105, Gaussian white noise is added to the ECG signal, for a signal-to-noise ratio (SNR) between 0.5 dB and 80 dB, and then the R-peak detection algorithm is applied. Record 102 is chosen because it is a relatively clean noise signal and record 105 is just the opposite. In this work, a comparison is made with the results from [10]. Gaussian white noise is added to the ECG signal with a certain signal-to-noise ratio according to the power of the ECG signal by means of the MATLAB command: y = awgn(y,SNR,‘measured’), where y is the ECG signal.

Table 9 shows the results for record 102. The sensitivity (S) starts to degrade from SNR = 60 dB with the algorithm of [10] and with the proposed algorithm, the degradation starts at SNR = 5 dB. As for the positive predictability (P), the results of [10] start to degrade at SNR = 40 dB and that of this work at SNR = 5 dB.

For a noisier record, 105, Table 10 shows that the sensitivity results of [10] degrade at SNR = 60 dB and at 20 dB for the proposed algorithm; in positive predictability, the results start to degrade at SNR = 40 dB in [10] and at 20 dB for this algorithm. In both tables, it is demonstrated that the proposed algorithm resists more noise before the performance indices start to degrade. It can also be observed that the proposed algorithm is more robust under the influence of noise.

## 6. Discussion

The proposed method utilizes single-parameter parabolic fitting, at each ECG sample, to extract a feature known as parabolic height. This feature detects partial P peaks, or candidates, by comparing them with a parabolic height threshold. Then, the best candidates are designated as R points of the ECG signal. Adapting such threshold to different ECG signal amplitudes is critical to the proposed method.

The algorithm designed for wearable devices requires low computational resources, because it does not need calculating and estimating the isoelectric line of the ECG signal, nor any prior filtering. The computational complexity of algorithms is persistent in using the number of registers and operations of addition, multiplication, and comparison. Compared to some of the more advanced portable implementations [5,13,15,21,22,23], the proposed algorithm is competitive.

The results derived from the application of the proposed algorithm to the QT database reveal that the performance of this algorithm is slightly superior with the state-of-the-art algorithms specialized in wearable devices [5,12,13] and demonstrate considerable competence, even in comparison with complex algorithms not specialized in wearable devices [3,8,9]. False negatives are primarily due to the R peak being broader and lower than most R peaks. False positives are mainly due to high-frequency noise.

We observe more modest results when evaluating the algorithm’s performance for the BIH-MIT database. The algorithm works with acceptable efficiency, except for the five most noisy records, where it yields many false positives and negatives due to the high noise level in these records. Nevertheless, considering that the ECG signals were recorded before 1989 [19] using older technology, a modern ECG signal acquired with current technology could be quieter than the mentioned records.

We perform two distinct types of analysis to assess the algorithm’s resilience against noise interference. First, we compare DER with other algorithms using six records that are notably susceptible to various forms of noise, including baseline drift and muscle noise. As employed in previous studies [5,24], these records serve as a standard for evaluating noise robustness.

Second, we subject R-peak detection to a rigorous test for the presence of white noise. To evidence the benefit of the proposed algorithm, Gaussian white noise is intentionally introduced to the ECG signal, with a specific signal-to-noise ratio determined by the characteristics of records 102 and 105, each with varying SNR levels. We then apply the R-peak detection algorithm to these modified signals. The results demonstrate that our proposed algorithm maintains its effectiveness in the presence of higher noise levels in both records before experiencing performance degradation. Furthermore, it is noteworthy that our proposed algorithm exhibits superior robustness when compared to the outcomes obtained in [10] under similar noisy conditions.

An area of opportunity that was detected in this work is that in most R-peak detectors, it is necessary to adjust the algorithm parameters to a different level. This can be resolved by establishing metaheuristic optimization strategies [31,32], which can be addressed in future work to adjust the algorithm parameters for different databases.

## Figures and Tables

**Table 1:** Computational complexity comparison.

Year	Method	Register	Adder	Multiplier	Comparator
2023	This algorithm	9	23	4	7
2019	Nguyen et al. [5]	4	11	4	1
2015	Castells and Carrabina [15]	41	8	1	14
2011	Wang et al. [21]	NA	14	5	15
2011	Elgendi [13]	6	15	11	4
2006	Hoang et al. [22]	21	23	1	1
1999	Jun et al. [23]	4	67	33	4

**Table 2:** Algorithm parameter values for simulations with the QT database.

Parameter	Value
$H_{min}$	0.3
$H_{max}$	1.1
$\alpha_{H}$	0.335
$n_{cand}$	70
$n_{th}$	480
L	17
M	4

**Table 3:** Performance evaluation of the proposed algorithm for the QT database.

Record	Total	TP	FN	FP	S (%)	P (%)	DER (%)	Record	Total	TP	FN	FP	S (%)	P (%)	DER (%)
sel100	1134	1134	0	0	100	100	0	sel16420	1063	1063	0	0	100	100	0
sel102	1088	1088	0	0	100	100	0	sel16483	1087	1087	0	0	100	100	0
sel103	1048	1048	0	0	100	100	0	sel16539	922	922	0	0	100	100	0
sel104	1109	1109	0	0	100	100	0	sel16773	1008	1008	0	1	100	99.90	0.10
sel114	862	862	0	4	100	99.54	0.46	sel16786	925	925	0	0	100	100	0
sel116	1185	1185	0	1	100	99.92	0.08	sel16795	761	761	0	0	100	100	0
sel117	766	766	0	0	100	100	0	sel17152	1628	1628	0	0	100	100	0
sel123	756	756	0	0	100	100	0	sel17453	1047	1047	0	0	100	100	0
sel213	1642	1641	1	0	99.94	100	0.06	sele0104	804	804	0	0	100	100	0
sel221	1247	1236	11	0	99.12	100	0.88	sele0106	896	896	0	3	100	99.67	0.33
sel223	1309	1308	1	0	99.92	100	0.08	sele0107	812	812	0	4	100	99.51	0.49
sel230	1077	1077	0	0	100	100	0	sele0110	872	872	0	4	100	99.54	0.46
sel231	732	732	0	0	100	100	0	sele0111	907	907	0	0	100	100	0
sel232	865	865	0	2	100	99.77	0.23	sele0112	684	684	0	1	100	99.85	0.15
sel233	1533	1532	1	0	99.94	100	0.07	sele0114	699	698	1	0	99.86	100	0.14
sel301	1351	1351	0	2	100	99.85	0.15	sele0116	558	558	0	4	100	99.29	0.72
sel302	1500	1500	0	1	100	99.93	0.07	sele0121	1436	1429	7	1	99.51	99.93	0.56
sel306	1040	1040	0	0	100	100	0	sele0122	1415	1414	1	0	99.93	100	0.07
sel307	853	853	0	0	100	100	0	sele0124	1121	1121	0	0	100	100	0
sel308	1294	1289	5	12	99.61	99.08	1.31	sele0126	945	945	0	1	100	99.89	0.11
sel310	2012	2012	0	0	100	100	0	sele0129	671	670	1	29	99.85	95.85	4.47
sel803	1026	1026	0	0	100	100	0	sele0133	840	840	0	0	100	100	0
sel808	903	903	0	3	100	99.67	0.33	sele0136	809	809	0	0	100	100	0
sel811	704	704	0	0	100	100	0	sele0166	813	813	0	0	100	100	0
sel820	1159	1159	0	0	100	100	0	sele0170	897	897	0	0	100	100	0
sel821	1557	1557	0	0	100	100	0	sele0203	1246	1246	0	6	100	99.52	0.48
sel840	1180	1180	0	0	100	100	0	sele0210	1063	1063	0	0	100	100	0
sel847	801	801	0	12	100	98.52	1.50	sele0211	1575	1575	0	0	100	100	0
sel853	1113	1113	0	0	100	100	0	sele0303	1045	1044	1	2	99.90	99.81	0.29
sel871	917	917	0	1	100	99.89	0.11	sele0405	1216	1216	0	0	100	100	0
sel872	990	990	0	0	100	100	0	sele0406	959	959	0	0	100	100	0
sel873	859	859	0	0	100	100	0	sele0409	1737	1737	0	0	100	100	0
sel883	892	892	0	1	100	99.89	0.11	sele0411	1202	1202	0	1	100	99.92	0.08
sel891	1267	1267	0	3	100	99.76	0.24	sele0509	1028	1028	0	0	100	100	0
sel14046	1260	1259	1	0	99.92	100	0.08	sele0603	870	869	1	0	99.89	100	0.11
sel14157	1081	1081	0	0	100	100	0	sele0604	1031	1031	0	0	100	100	0
sel14172	663	663	0	0	100	100	0	sele0606	1442	1442	0	0	100	100	0
sel15814	1036	1028	8	4	99.23	99.61	1.16	sele0607	1184	1184	0	1	100	99.92	0.08
sel16265	1031	1031	0	0	100	100	0	sele0609	1127	1126	1	0	99.91	100	0.09
sel16272	851	851	0	0	100	100	0	sele0612	751	751	0	0	100	100	0
sel16273	1112	1112	0	0	100	100	0	sele0704	1094	1093	1	1	99.91	99.91	0.18
Total									86,995	86,953	42	105	99.95	99.88	0.17

**Table 4:** Performance algorithms comparison for the QT database.

Year	Method	Total Beats	S (%)	P (%)	DER (%)
2023	This algorithm	86,995	99.95	99.88	0.17
2019	Nguyen et al. [5] *	85,353	99.94	99.78	0.29
2017	Pandit et al. [12] *	86,435	99.87	99.91	0.27
2015	Johnson et al. [8]	86,409	99.33	99.86	0.81
2013	Elgendi [13] *	85,353	99.76	99.42	0.82
2004	Martinez et al. [9]	86,892	99.92	99.88	0.20
1985	Pan and Tompkins [3]	85,353	99.60	98.35	2.07

* Algorithms for wearable devices.

**Table 5:** Algorithm parameter values for simulations with the BIH-MIT database.

Parameter	Value
$H_{min}$	0.45
$H_{max}$	0.9
$\alpha_{H}$	0.45
$n_{cand}$	115
$n_{th}$	691
L	35
M	4

**Table 6:** Performance evaluation of the proposed algorithm for the BIH-MIT database.

Record	Total	TP	FN	FP	S (%)	P (%)	DER (%)	Record	Total	TP	FN	FP	S (%)	P (%)	DER (%)
100	2273	2273	0	0	100	100	0	201	1963	1895	68	0	96.54	100	3.46
101	1865	1863	2	6	99.89	99.68	0.43	202	2136	2129	7	0	99.67	100	0.33
102	2187	2185	2	0	99.91	100	0.09	203	2980	2876	104	29	96.51	99	4.46
103	2084	2084	0	0	100	100	0	205	2656	2640	16	0	99.40	100	0.60
104	2229	2228	1	16	99.96	99.29	0.76	207	1860	1841	19	181	98.98	91.05	10.75
105	2572	2564	8	12	99.69	99.53	0.78	208	2955	2929	26	11	99.12	99.63	1.25
106	2027	2024	3	3	99.85	99.85	0.30	209	3005	3005	0	0	100	100	0
107	2137	2136	1	2	99.95	99.91	0.14	210	2650	2605	45	3	98.30	99.89	1.81
108	1763	1747	16	31	99.09	98.26	2.67	212	2748	2748	0	0	100	100	0
109	2532	2529	3	0	99.88	100	0.12	213	3251	3247	4	4	99.88	99.88	0.25
111	2124	2123	1	1	99.95	99.95	0.09	214	2262	2256	6	6	99.74	99.74	0.53
112	2539	2539	0	0	100	100	0	215	3363	3362	1	0	99.97	100	0.03
113	1795	1795	0	16	100	99.12	0.89	217	2208	2207	1	1	99.96	99.96	0.09
114	1879	1878	1	6	99.95	99.68	0.37	219	2154	2154	0	0	100	100	0
115	1953	1953	0	0	100	100	0	220	2048	2048	0	0	100	100	0
116	2412	2394	18	3	99.25	99.88	0.87	221	2427	2423	4	1	99.84	99.96	0.21
117	1535	1535	0	0	100	100	0	222	2483	2481	2	1	99.92	99.96	0.12
118	2278	2278	0	5	100	99.78	0.22	223	2605	2604	1	0	99.96	100	0.04
119	1987	1987	0	1	100	99.95	0.05	228	2053	2050	3	17	99.85	99.18	0.97
121	1863	1861	2	0	99.89	100	0.11	230	2256	2256	0	2	100	99.91	0.09
122	2476	2476	0	0	100	100	0	231	1571	1571	0	1	100	99.94	0.06
123	1518	1518	0	0	100	100	0	232	1780	1780	0	3	100	99.83	0.17
124	1619	1617	2	3	99.88	99.82	0.31	233	3079	3078	1	0	99.97	100	0.03
200	2601	2597	4	8	99.85	99.69	0.46	234	2753	2752	1	0	99.96	100	0.04
Total									109,490	109,120	373	373	99.66	99.66	0.68

**Table 7:** Performance algorithms comparison for the BIH-MIT database.

Year	Method	Total Beats	S (%)	P (%)	DER (%)
2023	This algorithm	109,490	99.66	99.66	0.68
2021	Bae et al. [10]	109,510	99.83	99.82	0.34
2019	Nyugen et al. [5] *	109,494	99.80	99.71	0.49
2016	Kim and Shin [14] *	109,494	99.90	99.91	0.19
2015	Castell-Rufas and Carrabina [15] *	109,494	99.43	99.67	0.88
2014	Dohare et al. [11]	109,966	99.21	99.34	1.45
1985	Pan and Tompkins [3]	116,137	99.76	99.56	0.68

* Algorithms for wearable devices.

**Table 8:** Comparisons of the DER from the proposed method with other studies for noisy records. The highest DER value in each column is shown in bold.

Methods	Year	Record					
		121	202	200	217	105	108
Proposed algorithm	2023	0.11	0.33	0.46	0.09	0.78	2.67
Nyugen et al. [5]	2019	0.16	0.09	0.35	0.09	1.21	1.53
Quadratic filtering [7]	2015	0	0	0.19	0.27	1.59	4.08
Wavelet transform [24]	2014	0.1	0.09	0.3	0.23	0.81	8.4
Elgendi’s algorithm [13]	2013	0.11	0.19	0.23	0.18	**1.87**	1.59
Linear filtering [25]	2013	0.11	0.09	0.15	0.09	1.25	0.57
S-transform [26]	2010	**0.16**	0.09	0.23	0.23	1.24	**2.44**
Artificial neural network [27]	2009	**0.16**	0.33	0.31	**0.64**	0.23	0.51
Mathematical morphology [28]	2014	0.7	**0.37**	0.5	0.23	1.01	0.68
Adaptive Mathematical morphology [29]	2016	0.11	0.09	0.19	0.45	1.44	1.13
Four QRS waveform templates [30]	2017	0.11	0.19	**0.73**	0.14	1.61	2.4

**Table 9:** Performance comparison for record 102 in [10] varying signal-to-noise ratio.

NSR (dB)	S (%) in [10]	S (%)	P (%) in [10]	P (%)
0.5	64.29	80.29	65.76	54.53
1	74.81	84.77	78.24	56.47
5	88.16	99.09	89.26	66.84
10	94.47	100	94.73	90.78
15	97.12	100	97.48	99.68
20	98.49	100	98.67	100
40	99.50	100	99.68	100
60	99.86	100	100	100
80	100	100	100	100

**Table 10:** Performance comparison for record 105 in [10] varying signal-to-noise ratio.

NSR (dB)	S (%) in [10]	S (%)	P (%) in [10]	P (%)
0.5	65.51	84.53	68.11	70.13
1	74.69	88.53	75.04	72.35
5	84.88	99.26	86.11	85.87
10	92.65	99.57	93.97	91.63
15	95.65	99.65	96.93	99.00
20	97.63	99.61	98.09	99.15
40	99.11	99.69	99.38	99.34
60	99.26	99.69	99.49	99.34
80	99.34	99.69	99.53	99.34

## Data Availability

Not applicable.
